# A cross-sectional study on the expression of fibrinolytic system components and metalloproteinase-9 in women with early-stage and metastases breast cancer in Tucumán, Argentina

**DOI:** 10.31744/einstein_journal/2025AO1237

**Published:** 2025-04-07

**Authors:** Mariano Nicolás Áleman, María Constanza Luciardi, Rosa Silvina Guber, Analía Graciela Soria

**Affiliations:** 1 Universidad Nacional de Tucumán Facultad de Bioquímica Cátedra Práctica Profesional Tucumán Argentina Cátedra Práctica Profesional, Facultad de Bioquímica, Universidad Nacional de Tucumán, Tucumán, Argentina.; 2 Universidad Nacional de Tucumán Facultad de Bioquímica, Química y Farmacia Cátedra Patología Molecular Tucumán Argentina Cátedra Patología Molecular, Facultad de Bioquímica, Química y Farmacia. Universidad Nacional de Tucumán, Tucumán, Argentina.

**Keywords:** Breast neoplasms, Breast disease, Metastasis neoplasms, Fibrinolysis, Matrix metalloproteinase, Standard coagulation tests, Plasminogen activator inhibitor 1

## Abstract

**Objective:**

The hemostatic and matrix metalloproteinase (MMP) systems are known to be altered in patients with breast cancer. This cross-sectional study aimed to explore the fibrinolysis components values and MMP-9 levels in a specific population of patients with early breast cancer and metastatic breast cancer in Tucumán, Argentina.

**Methods:**

This cross-sectional study included 63 patients without pathology or with benign breast pathology (GA Group), 48 patients with newly diagnosed primary breast cancer (GB Group), and 65 patients with metastatic breast cancer (GC Group). Levels of standard coagulation tests, tissue plasminogen activator (t-PA), plasminogen activator inhibitor-1 (PAI-1), D-dimer (DD), and matrix metalloproteinases-9 (MMP-9) were measured in all groups.

**Results:**

PAI-1 and MMP-9 levels were higher in patient with early breast cancer than those with benign disease. However, the GC Group showed significantly higher PAI-1, DD, and MMP-9 levels than the GA Group. Furthermore, the t-PA concentrations in the GC Group were lower than those in the Control Group.

**Conclusion:**

Our findings suggest that higher levels of PAI-1 and MMP-9 in patients with early-stage breast cancer may be associated with a subgroup of patients with more aggressive disease progression. In addition, significant alterations in t-PA, PAI-1, DD, and MMP-9 concentrations were observed in advanced stages of the disease. However, basic laboratory tests, such as global coagulation tests, do not effectively differentiate stages in breast cancer. Therefore, further study is needed to explore these findings in greater detail.

## In Brief

In this study, standard coagulation, t-PA, PAI-1, DD, and MMP-9 tests were performed in a specific population of patient with early and metastatic breast cancer in Tucumán, Argentina. Only PAI-1 and MMP-9 levels increased in patients with early-stage breast cancer, suggesting that they may have evolved aggressively.

## Highlights

■ No clinically significant differences were observed in platelet counts or global coagulation test results.
■ t-PA values decreased in patients with metastases.
■ PAI-1, DD, and MMP-9 levels increased in patients with metastases.
■ High levels of PAI-1 and MMP-9 in the early stages could indicate an aggressive course in patients.

## Graphical abstract

**Figure:**
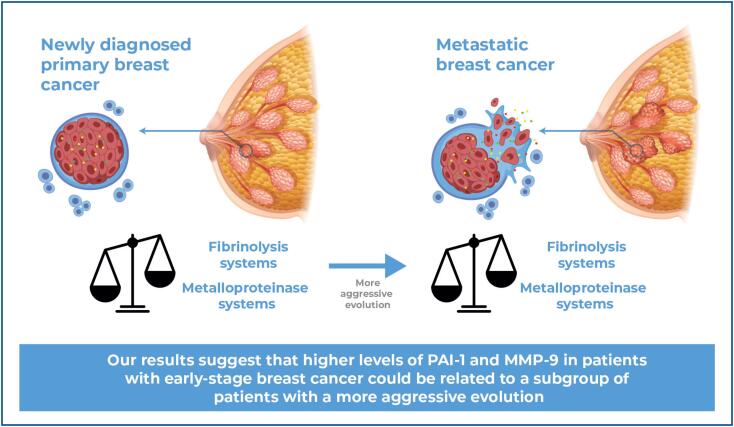

## INTRODUCTION

Breast cancer is the most common malignant tumor among women in Western countries, accounting for 25% of all female cancers in Argentina. In patients with breast cancer, coagulation regulatory mechanisms are altered, creating a prothrombotic microenvironment. The hemostatic system plays a critical role in processes associated with tumor progression such as extracellular matrix degradation, neo-angiogenesis, and distant metastasis.^(1)^ Among the mechanisms associated with the activation of the coagulation system in cancer, two procoagulant factors released by tumor cells have been reported: tissue factor, which initiates the clotting cascade and a cancer procoagulant that activates factor X. Literature supports the fact that the clinical and pathological characteristics of breast cancer correlate well with plasma hemostatic biomarkers. Additionally, studies have suggested an interaction between angiogenesis and hemostasis that promotes breast cancer metastasis.^(2)^

Fibrinolysis is a crucial physiological process that helps to maintain homeostatic balance by counteracting excessive thrombosis. Its components and their associations with various physiological and pathophysiological processes are well documented. Several fibrinolytic components have been shown to directly correlate with different steps in the tumor process.^(3)^ Therefore, fibrinolytic components are considered relevant in cancer biology, with studies suggesting that hemostatic elements can facilitate tumor metastasis, progression, proliferation, tumor cell survival, and angiogenesis in breast cancer.^(4)^

Several studies have been conducted to investigate molecules such as tissue plasminogen activator (t-PA), plasminogen activator inhibitor-1 (PAI-1), and D-dimer (DD), during cancer progression, evaluating their relationship with tumor size, and their usefulness as prognostic markers. Nevertheless, scientific evidence has shown inconsistency, with result varying across different populations.^(5,6)^ However, certain components of the fibrinolytic system have been suggested to directly associate with angiogenesis and neovascularization, thereby promoting the tumor. This information could enable each patient to be classified as high- or low-risk, aiding in personalized treatment a selection and adoption suitable for the risk.^(7)^ Accordingly, components such as t-PA, PAI-1, and DD are currently under investigation for clinical relevance.

Matrix metalloproteinases (MMPs) are a family of zinc-dependent endopeptidases involved in many physiological and pathological processes.^(8)^ Evidence suggests that MMPs are involved in the cleavage of extracellular matrix (ECM) and are closely related to tumor invasion and metastasis.^(9)^

In the tumor, matrix metalloproteinase-9 (MMP-9), once activated, can degrade and destroy type IV collagen and ECM gelatin near the tumor cell surface. This degradation allows tumor cells invade the surrounding basement membrane tissues, eventually leading to tumor invasion and metastasis. Additionally, MMP-9 influences the ability of tumor cells to adhere to tissues, thus playing an important role in promoting tumor growth and angiogenesis.^(10)^

Few studies in Latin America have examined hemostatic changes in patients with breast cancer; to date, no study has been conducted in the Argentinean region. Therefore, this study was conducted to provide valuable insight into the relevant characteristics of the target population, thereby providing information for improved treatment management of these patients. This cross-sectional study aimed to explore the standard coagulation tests, t-PA, PAI-1, DD, and MMP-9, in a population from Tucumán, Argentina, including individuals with benign breast pathologies, early-stage breast cancer, and metastatic breast cancer.

## OBJECTIVE

This cross-sectional study aimed to explore fibrinolysis components values and MMP-9 concentration in a specific population.

## METHODS

### Study population

This observational, analytical, and cross-sectional study was conducted at the Molecular Pathology Laboratory of Tucumán National University and the Maternity and Gynecology Institute in Tucumán City, Argentina, between January 2018 and March 2020. Participants with and without breast cancer were recruited for the study.

Study participants were categorized into following groups: GA: 63 patients without mammographically and clinically detectable pathologies or diagnosed with benign breast pathologies (fibrosis, adenosis, cysts), GB: 22 female patients with stage I breast cancer (no macroscopic involvement of lymph nodes or metastasis) and 26 with stage II (characterized by a larger tumor mass or axillary lymph node involvement); and GC: 65 patients with stage IV breast cancer. Clinical histories were obtained for all participants, including details on age, menopausal status, parity (nullity or low parity), history of breastfeeding, cigarette smoking status, previous breast diseases, and family history of breast cancer. The average ages were: GA 51.89±11.69; GB 52.3±12.0 and GC 53.7±11.2 years. Exclusion criteria for participants included preexisting systemic or focal inflammation, coagulopathy, and the use of medication such as anticoagulants and antiplatelet agents that could affect blood clotting and DD levels.

This study was conducted in adherence to the principles of the Declaration of Helsinki and Good Clinical Practice as defined by the International Conference on Harmonization. Ethical approval was granted by the Ethical Committee (CEI) of Tucuman University, Tucuman, Argentina (Ethical Committee N° 997/414-A). Written informed consents were obtained from all participants.

### Measurements of the study parameters

Venous blood samples were collected by venipuncture without the use of a tourniquet, between 8:00 and 10:00 AM. Platelet-poor plasma was obtained using different anticoagulants and prepared following the guidelines of the American Committee for Clinical Laboratory Standards and International Society for Hemostasis and Thrombosis. Coagulation parameters including prothrombin time (PT) activated partial thromboplastin time (aPTT), thrombin time (TT), and fibrinogen (Fg) levels were measured within 4h of sample extraction using an ACL 300 Coagulation Analyzer (Diamond Diagnostics, USA). Samples were aliquoted, carefully stored, and handled to prevent freeze-thaw cycles. Serum and venous blood samples were collected with EDTA disodium (10% w/v). Platelet count was performed using a Sysmex hematological counter (Sysmex KX-21, Kobe, Japan). PAI-1, t-PA, DD, and MMP-9 levels were determined using commercial enzyme-linked immunosorbent assay kits (Asserachrom PAI-1, Diagnostic Stago, Asnières sur Seine, France; Asserachrom t-PA, Diagnostic Stago, Asnières sur Seine, France; Asserachrom D-D, Diagnostic Stago, Asnières sur Seine, France; MMP-9 protein, R&D Systems Inc., Minneapolis, MN, USA).

### Statistical analysis

Statistical analysis was performed using IBM SPSS software version 25.0 (IBM Corp., Armonk, NY, USA). A descriptive analysis of the variables was performed. The Kolmogorov-Smirnov test was used to assess the distribution of the quantitative variables. Categorical data are expressed as frequencies and percentages while continuous variables are reported as mean ± standard deviation. Differences among groups were analyzed using one-way analysis of variance (ANOVA) with statistical significance set at p<0.05.

## RESULTS

### Clinic-pathological characteristics

Table 1 shows the clinic-pathological characteristics of patients in the GB and GC Groups. Infiltrating ductal adenocarcinoma was the most prevalent histological presentation in the breast cancer patient population, followed by invasive lobular carcinoma. The GC exhibited both bone and visceral metastases. Regarding hormonal receptor status, the proportions of estrogen-positive and progesterone-positive patients were 83.3%, 30.8%, and 62.1%, 23.1% for GB and GC, respectively. These differences were considered statistically significant; however, no significant differences were observed in HER2 expression between the GB and GC Groups.

**Table 1:** Clinic-pathological characteristics of the breast cancer subjects

Characteristics	GB Group (n=48)	GC Group (n=65)	p value
Age in years [X±SD]	52.3±12.0	53.7±11.2	0.521
Histological type, %			
IDC	66.6	72.3	0.060
ILC	25.0	27.7	
Other	8.3	0	
Stages of disease, %			
Stage I	45.8	-	
Stage II	54.2	-	-
Stage IV	-	100	
Receptor, %			
Estrogen (+)	83.3	30.8	0.001
Estrogen (-)	16.7	69.2	
Progesterone (+)	62.1	23.1	0.001
Progesterone (-)	37.9	76.9	
Her 2 (+)	21.2	32.3	0.176
Her 2 (-)	78.8	67.7	
Site of metastasis, %			
Bone	-	52.3	-
Lung	-	27.7	
Live	-	20.0	

GB: Group B; GC: Group C; IDC: invasive Ductal Carcinoma; ILC: invasive lobular carcinoma.

### Standard coagulation tests, t-PA, PAI-1, DD, and MMP-9

Table 2 shows the results of the hemostatic parameter assessments, including standard coagulation tests and concentrations of t-PA, PAI-1, DD, and MMP-9 in the different study groups. A significant difference in platelet count was observed among patients in GC Group compared to those in GA Group; while no significant differences were observed in the other values of the standard coagulation tests between the different groups. However, analysis of fibrinolysis parameters revealed that t-PA levels in patients with metastases significantly reduced compared to the Control Group (GA Group); whereas significant differences in PAI-1 and MMP-9 levels were observed between the GC *versus* GA Groups as well as between the GB and GA Groups. Notably, serum PAI-1 and MMP-9 levels were significantly elevated in patients with metastatic breast cancer compared to those with Stage I and II breast cancers. Additionally, the DD values were significantly increased in the GC Group compared to the remaining GA and GB Groups, and no differences were observed between the GA and GB Groups.

**Table 2:** Standard coagulation tests, t-PA, PAI-1, D-dimer and MMP-9

Parameters	GA Group (n=63)	GB Group (n=48)	GC Group (n=65)	p value
PT [% act]	90.88±7.54	91.67±5.38	91.6±7.86	0.930
aPTT [seg]	39.88±5.08	38.53±5.26	39.16±3.81	0.075
TT [seg]	21.41±0.61	21.67±1.04	21.58±0.83	0.127
Fg [ng/dL]	299.94±57.64	279.6±44.21	304.00±58.21	0.130
Platelet count [x10^9^/L]	209±44 *	256±92	266±14 *	0.018
t-PA [ng/mL]	0.56±0.33 *	0.46±0.21	0.40±0.21 *	0.004
PAI-1 [ng/mL]	1.87±0.72 *^†^	2.53±0.94 ^# †^	3.35±0.70 *^#^	0.001
D-dimer [ng/mL]	240±150 *	320±300 ^#^	800±250 *^#^	0.001
MMP-9 [ng/mL]	328±225 *^†^	433±103 ^# †^	929±103 *^#^	0.001

One-way ANOVA test (significant p<0.05).

* post-hoc analysis revealed significant difference between GC and GA;

# post-hoc analysis revealed significant difference between GC and GB;

† post-hoc analysis revealed significant difference between GA and GB.

GA: Group A; GB: Group B; GC: Group C; PT: prothrombin time; aPTT: activated partial thromboplastin time; TT: Thrombin time; Fg: Fibrinogen; t-PA: tissue plasminogen activator; PAI-1: Plasminogen activator inhibitor 1; MMP-9: metalloproteinase 9.

**Figure 1:**
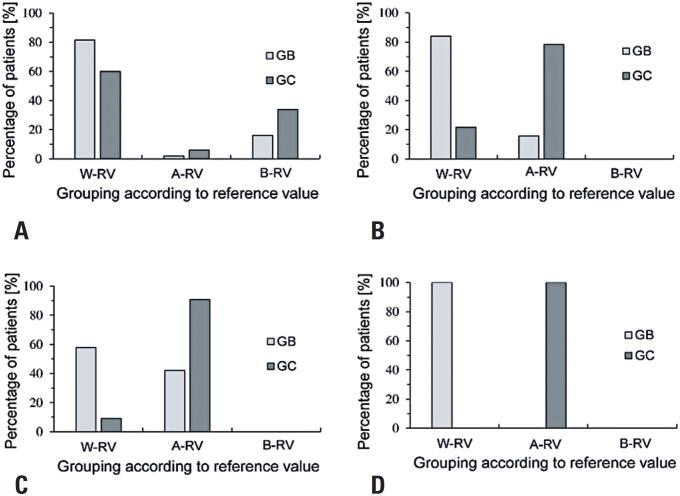
The chart shows the percentage of patients in the GB and GC Groups, categorized based on whether the molecule concentration is below, within or above the reference value for each molecule. The bars represent the percentage for: A) t-PA; B) DD; C) PAI-1; D) MMP9

GB: Group B; GC: Group C; W-RV: within the reference value; A-RV: above the reference value; B-RW: below the reference value; t-PA: tissue plasminogen activator; PAI-1: plasminogen activator inhibitor-1; DD: D-dimer; MMP9: matrix metalloproteinase-9.

The analysis of the percentage of patients belonging to GB and GC Groups, categorized based on the molecular values under study, revealed that the percentage of patients with metastasis and t-PA values below the reference value was higher than that of patients with an early diagnosis (GB= 16% *versus* GC=34%). Similarly, the percentages of patients with levels above the reference values for PAI-1 (GB= 42% *versus* GC= 91%), DD (GB= 16% *versus* GC= 78%), and MMP-9 (GB= 0% *versus* GC=100%) were predominantly from the GC Group. These findings highlighted the clear differences between the early-stage and metastatic breast cancer stages (Figure 1).

## DISCUSSION

Coagulation, fibrinolysis, and MMP systems plays pathophysiological roles in various processes, including tumor invasion and angiogenesis. The relationship between the fibrinolytic system and breast cancer has been investigated in many studies as cancer dissemination and a hypercoagulable state are leading causes of mortality in breast cancer. Therefore, researchers have suggested that endothelial dysfunction and consequent alteration of the fibrinolytic system are responsible for the migration of metastatic cells.^(11)^ This cross-sectional study aimed to explore standard coagulation tests, t-PA, PAI-1, DD, and MMP-9, in a population of Latin Americans with early breast cancer, metastatic breast cancer, and benign breast pathologies.

Our analysis of hemostatic parameters (PT, aPTT, TT, and Fg) did not show any significant differences between the groups. However, platelet counts were higher in patients with metastases than in the other groups. Previous studies have shown thrombocytosis to be associated with poor prognosis, suggesting a direct role of platelets in the disease pathogenesis.^(12)^ A study conducted by Dirix et al. measured PT and platelet counts in patients with metastatic breast cancer (n=84) and newly diagnosed women (n=23) and did not find significant differences between the groups. However, significant changes in Fg levels were observed.^(13)^ Whereas Mi et al. observed that PT and aPTT were decreased and Fg levels were increased in patients with breast cancer.^(14)^ Moreover, a French study conducted by Chaari et. al involving 62 patients with breast cancer at various stages investigated several biomarkers of hypercoagulability and found no significant differences in PT, aPTT, TT, or Fg values between the groups.^(15)^ In contrast, Tas et al. evaluated the correlation between blood coagulation assays in patients with breast cancer and healthy individuals, identifying differences in aPTT, platelets counts, and Fg levels, but not in PT.^(16)^ These divergences between studies may be due to the sensitivity of the reagents used for the detection of these parameters, as well as to the specific conditions of the population studied or the underlying biology of the cancer itself.

An interesting finding of this study was the decreased t-PA concentrations in women with metastatic breast cancer compared to those in the Control Group; these differences were significant but did not differ from those in early-stage breast cancer. These findings are consistent with those reported by Wrzeszcz et al., who analyzed fibrinolytic factors to assess the risk of breast cancer dissemination and found that low levels of t-PA antigen could be used as an independent biomarker for early metastasis risk and were associated with reduced disease-free survival.^(17)^ Similarly, Corte et al. observed comparable results in homogenized tumor extracts from patients with breast cancer, in which they measured t-PA levels and noted that low values were associated with better overall survival.^(18)^ However, other researchers have reported positive associations between cancer progression and serum t-PA levels; which means that higher plasma t-PA levels are associated with an aggressive form of breast cancer due to the degradation of the extracellular matrix enhanced by hyperfibrinolysis.^(19)^ A recent study performed in cell culture and orthotopic mammary fat pad tumor xenograft experiments in rats explored the regulation of t-PA gene expression and found that increased t-PA expression plays a key role in breast cancer progression.^(20)^ The inconsistency in the results may be due to the absence of standard cutoff points for fibrinolytic factors in patients with breast cancer. Additionally, plasmin activation by factors other than t-PA (*e.g*., uPA) should be considered as contributing mechanism.

Regarding PAI-1 levels, we found an increase in patient with metastatic breast cancer compared to those with early-stage breast cancer and benign pathologies. However,, participants with stages I and II breast cancer showed significantly higher values than those in the Control Group. These findings are consistent with those of several other studies suggesting that elevated intratumoral PAI-1 levels and other components of the fibrinolytic system are independent predictors of impaired survival outcomes in patients with breast cancer.^(21-23)^ A 10-year prospective biomarker-based trial demonstrating the prognostic utility of PAI-1 in breast cancer and defining the level of evidence (LoE-1) concluded that high PAI-1 levels in both lymph node-positive and lymph node-negative patients were independently associated with poor prognosis.^(24)^ Furthermore, recent studies have suggested that PAI-1 plays a role in the metastatic potential of cytokeratin-positive (dCK+) cells and enhances the potential of individual proteins to attract protumor neutrophils, highlighting its critical role in breast tumor progression.^(25,26)^

Our findings on serum DD revealed that patients with advanced cancer had significantly higher levels than those with early stage breast cancer and the Control Group, with no notable differences between the latter two groups. Therefore, this findings suggest that DD may serve as a marker indicative of metastatic disease progression. This agrees with the findings published by Sringeri et al., which evaluated the role of DD in breast carcinoma patients to predict metastasis. Their study tested DD levels before treatment and after 6 months, showing a direct correlation with lymph node involvement and invasion.^(27)^ Other research have reported significant increases in DD concentration in patients with breast cancer compared to healthy patients and a tendency to increase in advanced stages of the disease. Additionally, DD levels were higher in the metastatic stage than in the local stage and increased further in the subgroup of patients with metastases following chemotherapy.^(28,29)^ However, a study that included 788 patients with breast cancer evaluated hemostatic biomarker alterations prior to chemotherapy and found that high levels of DD were significantly associated with lymph node metastasis.^(30)^

Finally, our analysis of MMP-9 showed significantly elevated levels of concentration in newly diagnosed patients compared to those in Control Group. Similarly, patients with metastatic breast cancer exhibited significantly increased levels of MMP-9 than early-stage patients and the Control Group. These findings are consistent with majority of published studies conducted globally. Furthermore, Patel et al. studied MMP-2 and -9 as serum prognostic biomarkers in a cohort of 60 women with primary breast cancer, 40 with benign breast disease, and 60 healthy controls in India. They observed a significant increase in serum MMP-9 levels in patients with metastases and concluded that serum MMP-9 is a better marker than serum MMP-2 for predicting the development and progression of breast cancer.^(31)^ Additionally, other authors in India reported higher mRNA expression levels of MMP-9 and vascular endothelial growth factor (VEGF-C) in breast cancer biopsy specimens compared to benign breast disease. and could act as markers of tumor presence. Additionally, MMP-9 and VEGF-C expression were significantly associated with lymph node status, highlighting their potential as valuable diagnostic marker for lymph node metastasis in patients with breast cancer. Notably, MMP-9 expression was associated with tumor size.^(32)^ A study conducted in Ethiopia analyzed the expression of MMP-2, MMP-9, and MMP-11 in breast tissue samples from patients with benign tumors and breast cancer. The authors concluded that MMP-11 expression was higher in breast cancer tissues than in benign tumors. Furthermore, higher levels of MMP-11, MMP-9, and MMP-2 were observed in tissues from breast cancer patients with lymph nodes and estrogen receptor-positive, suggesting the critical role of MMPs in the pathophysiology of breast cancer.^(33)^ Similarly, a recent study conducted in Egypt investigated the roles of MMP-2 and MMP-9 in women with breast cancer by measuring their plasma concentrations preoperatively and postoperatively. The findings revealed that plasma levels of MMP-2 and MMP-9 significantly increased for all TNM tumor stages prior to the curative surgery and decreased significantly following surgery.^(34)^ This study, in agreement with many other studies, suggests that elevated MMP-9 expression may indicate the presence of breast cancer.

### Limitations of the study

This study had certain limitations. It was designed as a cross-sectional study, with the corresponding potential design bias and a small sample size; however, the molecular levels assayed in each participant were measured using a single plasma sample collected at the time of recruitment of the population. Finally, it is essential to establish well-defined cutoff points for t-PA, PAI-1, DD, and MMP-9 in Latin American women, as these parameters may vary across populations.

## CONCLUSION

One of the strengths of this study lies in its contribution to the limited data available on molecular changes in population across different stages of breast cancer in summary, our results suggest that despite the small sample size, higher levels of PAI-1 and MMP-9 in patients with early-stage breast cancer could be related to a subgroup of patients with a more aggressive evolution. Future research should include longitudinal studies and the determination of these biomarkers. Additionally, t-PA, PAI-1, DD, and MMP-9 concentrations are significantly altered in advanced stages of the disease. Basic laboratory tests such as global coagulation assays are insufficient to distinguish between stages in patients with breast cancer. Further studies are warranted to better understand these biomarkers and their role in breast cancer progression.
